# Advances in nanomaterials

**DOI:** 10.3762/bjnano.4.91

**Published:** 2013-11-27

**Authors:** Herbert Gleiter, Horst Hahn, Thomas Schimmel

**Affiliations:** 1 Institute of Nanotechnology, Karlsruhe Institute of Technology, 76021 Karlsruhe, Germany

In many cases new technologies are driven by the development of novel materials providing properties or combinations of properties that were not available before. The use of polymers and silicon-based semiconductor technologies are just two examples of how the availability of certain materials revolutionized existing technologies and products, giving rise to completely new technologies and having an enormous impact on our everyday life.

During the past three decades, the field of nanomaterials experienced a tremendous rise from the early beginnings to modern-day technologies, and still, new ideas and breakthroughs keep opening new perspectives for future technologies, from cars to aircraft, from semiconductors and electronics to novel displays, from energy harvesting to energy storage technologies and from biomimetic structures to medical technologies, to mention just a few examples.

One recent development in the field of nanomaterials is the ability not only to tailor the properties of nanomaterials (to achieve custom-designed, “tailor-made” values of certain properties) but to *tune* these properties. Tunable materials allow to change the properties of these materials *reversibly and in a controlled manner* after fabrication, e.g., by applying an electric field. Another remarkable development is the discovery of *“nanoglasses”,* based on the idea of introducing internal interfaces on the nanometer scale not between crystalline structures (leading to nanocrystalline materials) but between non-crystalline, amorphous or glassy structures. These glasses differ structurally from present-day glasses and thus are expected to open the way to an age of glass-based technologies comparable to the contemporary, primarily crystalline-based, technologies.

This Thematic Series does not attempt to give a comprehensive overview over the vast and thriving field of nanomaterials. It rather gives a sequence of contributions on recent developments within this field.

The volume is dedicated to Horst Hahn on the occasion of his 60th birthday. Horst Hahn is Executive Director of the Institute for Nanotechnology at the Karlsruhe Institute of Technology (KIT) and Full Professor at the Technische Universität Darmstadt. In addition, he is Founding Director of the Helmholtz Institute Ulm for Electrochemical Energy Storage (HIU). He is Distinguished Professor at the Indian Institute of Technology Madras, India, and Honorary Professor at the University of Hyderabad, India, and at the Lanzhou University, China. Moreover, he is Elected Member of the German Academy of Sciences Leopoldina and the European Academy of Science. As a Fellow of the Materials Research Society (MRS), his sustained and distinguished contributions to the advancement of international materials research were recognized. In the past two years, Horst Hahn was recipient of the highest award of the Deutsche Gesellschaft für Materialkunde (DGM), the Heyn Denkmünze, and of the renowned Robert Franklin Mehl Award of the Minerals, Metals & Materials Society (TMS). Horst Hahn has published more than 300 peer-reviewed articles in various fields of materials science. His main research interests are related to the synthesis and processing of nanocrystalline materials and nanostructures with tailored and tunable properties, with a focus on applications in printed electronics and energy materials.

The nucleation point for this Thematic Series was an international symposium held in August 2012 at the Karlsruhe Institute of Technology on the occasion of the 60th birthday of Horst Hahn – the “Nanomaterials Days” – which brought together renowned experts in the field of nanomaterials, each of them giving a short contribution on one recent research highlight in this field. A selection of these contributions is found in this Thematic Series in the form of original research articles reflecting recent advances in nanomaterials.

The articles in this Thematic Series highlight recent developments, from nanoporous polymers to graphene quantum dots, from concepts for designing magnetic properties to nanoplasticity and to the remarkable mechanical properties of a novel type of nanowires, just to mention some of the developments described.

We would like to thank all the authors for contributing their excellent work to this Thematic Series. Moreover, we would like to thank all referees for their promptly provided reports keeping the publication times short and attractive for contributors. Finally, we thank the team at the Beilstein Institut for their excellent support, and we acknowledge to the open access policy of the Beilstein Journal of Nanotechnology, which provides the basis for unrestricted discussions on “Advances in Nanomaterials”.

Herbert Gleiter, Horst Hahn and Thomas Schimmel

Karlsruhe, November 2013

